# Multivariate Analysis Models Based on Full Spectra Range and Effective Wavelengths Using Different Transformation Techniques for Rapid Estimation of Leaf Nitrogen Concentration in Winter Wheat

**DOI:** 10.3389/fpls.2020.00755

**Published:** 2020-06-26

**Authors:** Lantao Li, Di Lin, Jin Wang, Liu Yang, Yilun Wang

**Affiliations:** ^1^College of Resources and Environment, Henan Agricultural University, Zhengzhou, China; ^2^College of Forestry, Henan Agricultural University, Zhengzhou, China; ^3^Soil and Fertilizer Station of Jiaozuo City, Jiaozuo, China

**Keywords:** precision nitrogen management, spectral analysis, estimation model, first derivative reflectance, partial least square regression

## Abstract

To develop a stable estimation model and identify effective wavelengths that could explain the variations in leaf nitrogen (N) concentration with different N supplies, growing seasons, ecological locations, growth stages, and wheat cultivars. Four field experiments were performed during two consecutive years (2017–2019) at three sites (Yuanyang, Hebi, and Wenxian) in Henan, China. *In situ* canopy spectral reflectance data under the aforementioned N supply conditions were obtained over a range of 400–950 nm (visible and near-infrared region). On the basis of the canopy raw spectral reflectance data and their subsequent transformation by two different techniques, first-derivative reflectance (FDR) and continuum removal (CR), four multivariate regression methods were comparatively analyzed and used to develop predictive models for estimating leaf N concentration: multiple linear regression (MLR), principal component regression (PCR), partial least square (PLS), and support vector machine (SVM). Results showed that leaf N concentration and canopy reflectance significantly varied with the levels of N fertilization, and a good correlation was observed for all the spectral techniques. Seven wavelengths with relatively higher *r* values than the bands of the raw spectra centered at 508, 525, 572, 709, 780, 876, and 925 nm were specified using the FDR technique. Based on the full wavelengths, the FDR-SVM model exhibited a good performance for leaf N concentration estimation, with coefficients of determination (*r*^2^_val_) for the validation datasets and corresponding relative percent deviations (RPD_val_) values of 0.842 and 2.383, respectively. However, the FDR-PLS yielded a more accurate assessment of the leaf N concentration than did the other methods, with *r*^2^_val_ and RPD_val_ values of 0.857 and 2.535, respectively. The variable importance in projection (VIP) scores from the FDR-PLS with the all canopy spectral region were used to screen the effective wavelengths of the spectral data. Therefore, six effective wavelengths centered at 525, 573, 710, 780, 875, and 924 nm were identified for leaf N concentration estimation. The SVM regression method with the effective wavelengths showed excellent performance for leaf N concentration estimation with *r*^2^_val_ = 0.823 and RPD_val_ = 2.280. These results demonstrated that the *in situ* canopy spectral technique is promising for the estimation of leaf N concentration in winter wheat based on the FDR-PLS regression model and the effective wavelengths identified.

## Introduction

Nitrogen (N) is an essential element of pigments as well as proteins associated with crop N status, and N is important in terms of plant vigor, yield formation, and grain quality (Din et al., 2018). Precise N management and accurate estimation of crop N status are the most common problems in modern agricultural systems not only for economic reasons but also for minimizing the atmospheric, soil, and water pollution associated with excessive N supply (Zhao et al., 2016; Din et al., 2018). Currently, several techniques for the non-destructive estimation of crop N have been proposed, including leaf color charts, SPAD-502, Dualex 4, and CCM-2000 (Schächtl et al., 2005; Din et al., 2018; Zhao et al., 2018). However, all these instruments center on the local test of the leaves and are not practical for application across large fields. Moreover, the techniques actually rely on the absorption of crop chlorophyll and carotenoid instead of N. Several elements, such as leaf thickness, leaf specific mass, the leaf position, the areas from which leaves are measured (Daughtry et al., 2000; Muñoz-Huerta et al., 2013), crop growth, cultivar, and solar radiation (Tahir Ata-Ul-Karim et al., 2016; Din et al., 2018), can influence the results. To overcome these problems, canopy spectral remote sensing (CSRS) has emerged and is recommended as an alternative effective and non-destructive technique for rapidly estimating crop N status (Yao et al., 2015; Prey and Schmidhalter, 2019).

Canopy spectral remote sensing is a promising approach for the accurate and real-time estimation of crop N status and other growth variables over large areas (Ecarnot et al., 2013; Feng et al., 2015). CSRS analyses may be performed with field-based spectral radiometers such as the ASD FieldSpec Handheld 2, which can generate a high resolution (<5 nm) and continuous spectrum at each pixel that is influenced by N compounds, chlorophyll status, and crop structures; therefore, it provides an effective method for assessing leaf N concentration at whole canopy scales (Gómez-Casero et al., 2010; Yao et al., 2010; Feng et al., 2015). However, the canopy raw spectra are, however, influenced by the solar radiant flux, crop structure characteristics (e.g., biomass, leaf area index, blade incidence, and plant height), and soil background conditions (Feng et al., 2014; Mahajan et al., 2014). Thus, identifying effective wavelengths for rapidly estimating crop leaf N concentration has become an extremely important topic in canopy spectral studies. Several canopy spectral transformation techniques, such as first-derivative reflectance (FDR) (Ihuoma and Madramootoo, 2019; Wen et al., 2019) and continuum removal (CR) (Tian et al., 2017; Tan et al., 2019), have been used to improve the signal-to-noise ratio, minimize the impact of atmospheric noise, and enhance weak spectral information of remote monitoring of leaf N concentration in crops. Experimental investigations have shown that the FDR technique can resolve overlapping absorption phenomena and can minimize the influences of soil or atmospheric background noise (Hruschka, 1987; Miphokasap et al., 2012). Moreover, the CR technique can smooth the spectra, eliminate signal errors caused by the instruments themselves, and suppress the noise within spectral data (Mutanga et al., 2005; Summers et al., 2009; Tan et al., 2019).

On the basis of nearly contiguous spectral wavelengths, overfitting, redundancy, and multicollinearity problems might occur during the modeling of the canopy raw spectra and their subsequent transformation (e.g., *via* FDR or CR). Multivariable statistical regression methods such as multiple linear regression (MLR), principal component regression_(PCR), and partial least square (PLS) analysis were used to reduce multicollinearity and establish a quantitative monitoring model for estimating leaf N concentration (Hansen and Schjoerring, 2003; Wang et al., 2011; Thorp et al., 2017). Rather than using individual wavelengths for the construction of vegetation indices, the aforementioned approaches incorporate all wavelength data into models for the estimation of plant physiological and biochemical properties (Thorp et al., 2017). For example, MLR is the linear combination of the full-range spectral reflectance and is also the most widely used method for rapidly estimating crop leaf N concentration using spectral measurements (Huang et al., 2004; Wang et al., 2011). The PCR is a linear regression that first decomposes the spectra into a suite of PCs that offers the maximum variation of the spectra with the aim of optimizing the estimative capacity of the model; it then regresses the PCs against the response variable (Cho et al., 2007; Foster et al., 2016). PLS is closely related to PCR. The difference between PCR and PLS is that while the former uses only the independent variables (e.g., spectral wavelengths) to construct new PCs, the PLS uses both the independent and dependent variables (e.g., leaf N concentration) that will play the role of explanatory variables to construct PCs. Moreover, the PLS method can reduce the high dimensional and collinear spectral reflectance data to a small quantity of latent variables and effectively eliminate or minimize the overfitting problem (Foster et al., 2016). However, MLR, PCR, and PLS were initially selected for laboratory spectroscopy but are now increasingly used for analyzing CSRS data of maize (Weber et al., 2012; Kawamura et al., 2018), rice (Inoue et al., 2012; Li F. et al., 2014; Li X. C. et al., 2014), and grasslands (Kawamura et al., 2010). Previous studies showed that the selection of effective wavelengths can refine the predictive ability of the standard full spectrum by optimizing important effective wavelengths; therefore, several effective wavelength selection methods such as MLR, PCR, and PLS have been developed (Bolster et al., 1996; Yao et al., 2015; Kawamura et al., 2018). Nonetheless, only a limited number of studies made an attempt to evaluate the aforementioned effective wavelength selection methods in combination with spectral transformation techniques (CR and FDR) for a comparative and comprehensive estimation of leaf N concentration of winter wheat. Moreover, limited previous research, along with its recommended algorithms and spectral indices, has been conducted to estimate the leaf N concentration in the same ecological locations (Montes et al., 2011; Cao et al., 2017; Zhao et al., 2018; Bruning et al., 2019), neglecting the problem of unsynchronized winter wheat growth stages under different conditions. Therefore, further work is needed to systematically analyze the performance of multiple methods for predicting leaf N concentration in winter wheat under different ecological areas, unsynchronized growth stages, cultivars, and N supply.

The specific objectives of this study were to (1) study the relationship between winter wheat leaf N concentration and the *in situ* canopy raw spectra and the transformation techniques from field spectral data; (2) compare the reliability and performance of the applied multivariate regression methods based on the raw and the transformed (FDR, CR) spectra for the estimation of leaf N concentration; (3) determine the optimal method with highest robustness and accuracy and lowest complexity that can rapidly estimate the leaf N concentration of winter wheat; and (4) determine the sensitivity of the effective wavelengths by using the identified monitoring method and construction of an estimation model of wheat leaf N concentration.

## Materials and Methods

### Experimental Design

**FIGURE 1:**
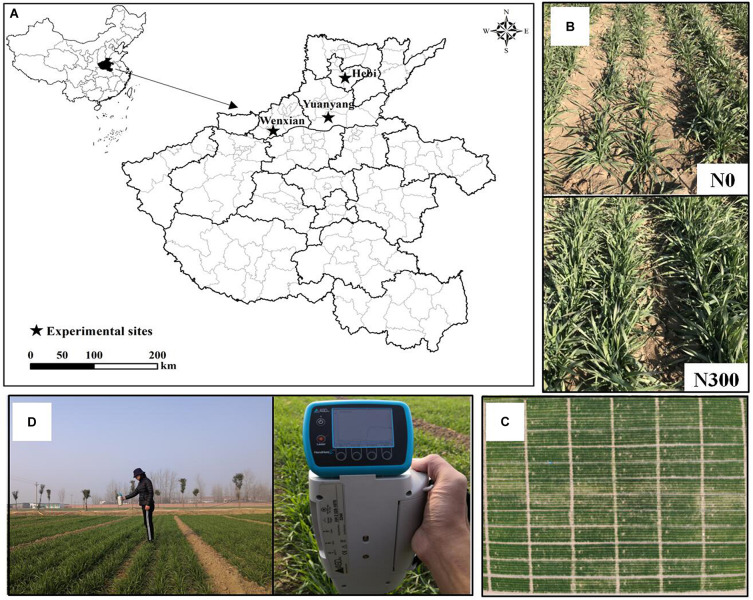
Sites **(A)**, close-up view **(B)** of the field experiments, aerial view of the N supply plots **(C)**, and measurements of winter wheat canopy spectral reflectance **(D)**.

Four experiments on wheat were carried out across two growing seasons, with one field located in Yuanyang County (35°6′ N, 113°56′ E), two in Hebi city (35°40′ N, 114°17′ E), and one in Wenxian County (34°57′ N, 112°59′ E) in Henan Province, North China (Figure 1A). The following variables were included in the study of hexaploid winter wheat: year, ecosystem, cultivar, N application rate, and sampling date. A randomized complete block design including all treatments in the field experiments was applied, with three replications (Supplementary Table S1 and Figures 1B,C). For all the treatments, phosphorus (P) and potassium (K) nutrition were applied. The recommended P and K fertilizer rates were 120 kg ha^–1^ (as superphosphate, 12% P_2_O_5_) and 90 kg ha^–1^ K_2_O (as potassium chloride, 60% K_2_O). Moreover, the detailed information of N supply (as controlled-release urea, 44% N) is shown in Supplementary Table S1. All nutrient resources were applied as a basal fertilizer prior to sowing. Other winter wheat management practices, such as the use of herbicides and disease and pest control followed the local standard practices during the two growing seasons.

### Sampling and Measurement

#### Spectral Measurements

In this study, all the *in situ* canopy reflectance spectra were obtained with an ASD FieldSpec Handheld 2 passive spectroradiometer (ASD Inc., Boulder, CO, United States) at nadir from a height of approximately 1.0 m above the winter wheat canopy under sunny conditions between 11:00 and 14:00 (Figure 1D). To reduce the influence of atmospheric and field conditions, the winter wheat canopy spectral reflectance was measured at six sites in each plot, and 60 scans served as the mean canopy spectrum for each plot. A 30 × 30-cm BaSO_4_ calibration Spectralon^®^ panel (Spectralon^®^, Labsphere, Inc., North Sutton, NH, United States) was applied to calibrate the reflectance and radiance before and after taking a measurement. Wavelengths below 400 and above 900 nm were excluded due to the low signal. Therefore, the canopy reflectance data were resampled within the range of 400–900 nm.

#### Leaf Nitrogen Concentration

After each measurement of *in situ* canopy spectral reflectance, four areas of 0.30 m^2^ (60 cm long × 50 cm wide; the spacing interval between the two rows was 20 cm) of winter wheat plants from each plot were immediately selected to determine the leaf N concentration (%) values by the H_2_SO_4_-H_2_O_2_ method (Thomas et al., 1967). The leaf N concentration was measured *via* a flow injection auto-analyzer (AA3, Bran and Luebbe, Norderstedt, Germany).

### Transformation Techniques of Winter Wheat *in situ* Canopy Spectra

To smooth the spectra, the frequently used Savitzky-Golay filter was applied, and a second-order polynomial with a window size of five spectral wavelengths was added to eliminate signal noise (Gholizadeh et al., 2015). Afterward, two spectral transformation techniques, FDR and CR, were compared to identify the best techniques for the rapid estimation of the leaf N concentration from the raw reflectance spectra (Curran et al., 2001; Sims and Gamon, 2002).

### First-Derivative Reflectance

First-Derivative Reflectance spectral transformation technique was applied to reduce the impacts of multiple scattering of radiation (Ihuoma and Madramootoo, 2019). The FDR formula is as follows (Miphokasap et al., 2012):

(1) $$\mathrm{FDR}(\lambda_{\text{i}}=\frac{\left[R\,\left(\lambda_{i+1}\right)-R\,\left(\lambda_{i-1}\right)\right]}{2\,\Delta \,\lambda }$$

Where R(λ_*i*__+__1_) and R(λ_*i*__–__1_)are the reflectance values at i + 1 and i−1, respectively; and Δλ is the wavelength increment.

### Continuum Removal

**FIGURE 2:**
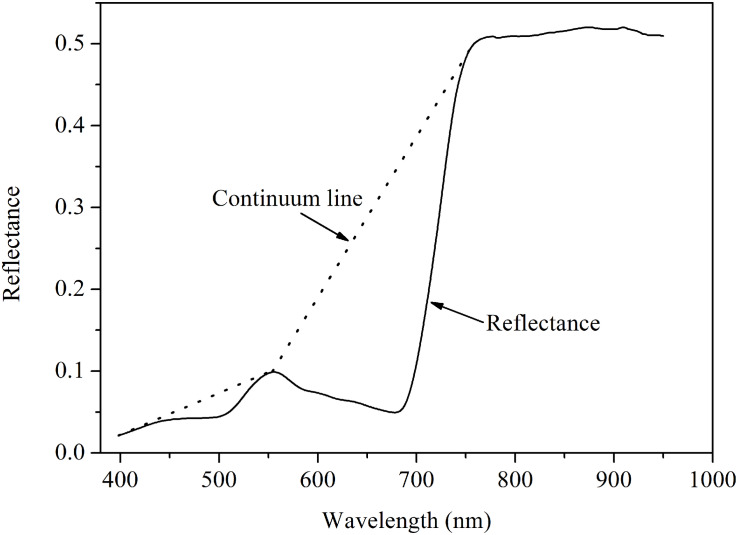
Schematic diagram of the continuum removal technique on the winter wheat canopy.

The CR spectral transformation technique was also used to estimate the leaf N concentration. The continuum line is a convex hull to connect the local maxima of a spectrum (Figure 2). The method was used to assess crop biochemicals with dried plant leaves (Curran et al., 2001), and to the best of our knowledge, studies that have extended this technique to *in situ* canopy spectra under field conditions combined with the quantification of leaf N concentration in winter wheat are relatively rare (Li et al., 2019).

### Data Analysis

Four different multivariate regression methods were comparatively analyzed and used to estimate the winter wheat N status at the canopy levels: MLR, PCR, PLS, and support vector machine (SVM).

### Multiple Linear Regression Analysis

Multiple linear regression is a common approach used to calibrate the relationship between multiple independent variables and a dependent variable, which was successfully used for the evaluation of *in situ* canopy spectra and involved stretching the results of a simple linear regression analysis from a single dimension into multiple dimensions (Bruning et al., 2019).

### Principal Component Regression Analysis

Principal component regression is based on principal component analysis and is also a widely adopted method for dimensionality reduction of spectral data. The optimal number of components to contain in the model was identified by the number of components that had the lowest root mean square error of cross-validation (RMSECV). A 20-fold leave-one-out cross-validation procedure was applied for validating the PCR models to avoid overfitting or underfitting.

### Partial Least Square Analysis

Partial least square is a powerful method that can be used to reduce the *in situ* reflectance data effectively into a few latent variables with information content and thus maximize the covariance between the spectra and leaf N concentration. The optimal number of latent variables (ONLVs) of the PLS was the same as that of the PCR and was confirmed based on the lowest RMSECV using the leave-one-out method. In addition, reflectance spectra obtained in this study contained 551 bands in the range of 400–950 nm. The variable importance in projection (VIP) scores resulting from the PLS were applied to select the effective wavelengths for rapidly estimating the leaf N concentration. The threshold score of a VIP is 1.0; therefore, a higher VIP score indicates that the wavelength is more important to estimate the leaf N concentration, while the wavelength having a lower VIP score has less impact on the estimation (Word et al., 2001).

### Support Vector Machine Analysis

In this study, the Gaussian radial basis function (RBF) kernel was used for the SVM technique, and the detailed introduction of the SVM regression model is reported by Schölkopf et al. (2000) and Bishop (2006).

### Model Calibration and Validation

The data function from the four field experiments is shown in Supplementary Table S1. Correlation analysis between the *in situ* reflectance spectra and leaf N concentration was performed using the SAS 8.0 (SAS Institute, Cary, NC, United States). The MLR, PCR, PLS, and SVM methods were analyzed using MATLAB 2019a (MathWorks, Natick, MA, United States). The performance of all the regression models was evaluated by the coefficient of determination (*r*^2^), root mean square error (RMSE), and relative percent deviation (RPD) in both the calibration and validation datasets. Detailed information concerning the *r*^2^ and RPD values of the regression methods is shown in Table 1 (Chang et al., 2001).

**TABLE 1:** Classification of the performance of the regression methods in terms of *r*^2^ and RPD values.

Standards	Model performance		
	Unacceptable	Acceptable	Excellent
*r*^2^	<0.50	0.75–0.50	>0.75
RPD	<1.40	2.00–1.40	>2.00

*RPD, relative percent deviation.*

## Results

### Leaf Nitrogen Concentration in Winter Wheat

Table 2 shows the results of the descriptive analyses of the leaf N concentration in the calibration, validation, and combined datasets. In the combined datasets, the treatments (N rates, ecological sites, and growing seasons and stages) generated a wide range of leaf N concentration (1.06–6.16%). Among the datasets, compared with those of validation datasets, the mean and range of the calibration datasets were greater, showing that the classification of the data is appropriate.

**TABLE 2:** Statistics of winter wheat leaf nitrogen concentration for the calibration and validation datasets.

Datasets	Number of samples	Mean (%)	Maximum (%)	Minimum (%)	SD	CV (%)
Calibration datasets	165	3.90	6.16	1.06	1.21	31.03
Validation datasets	150	3.70	6.02	1.12	1.27	34.32
All datasets	315	3.80	6.16	1.06	1.24	32.63

*SD, standard deviation; CV (%), coefficient of variation.*

### Variability of the *in situ* Spectra Obtained at Various Nitrogen Supplies

**FIGURE 3:**
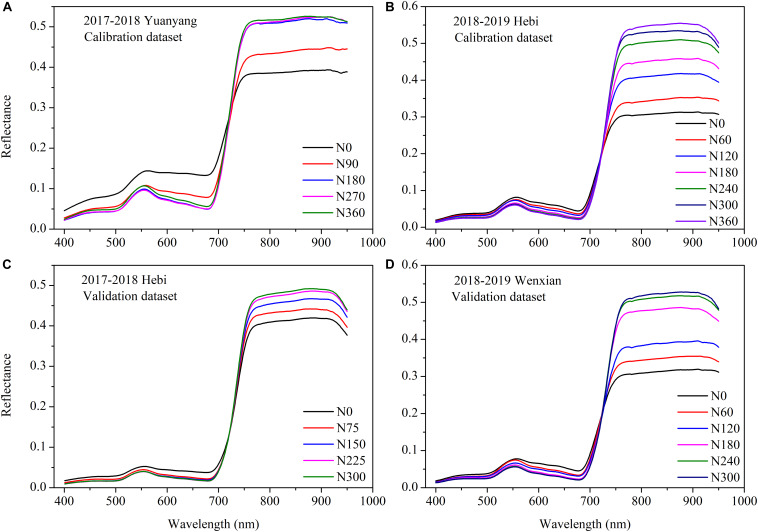
Effect of nitrogen (N) status on the *in situ* canopy spectral reflectance of winter wheat for the calibration **(A,B)** and validation **(C,D)** datasets.

The spectral characteristics of canopy reflectance were significantly different under the different N treatments but exhibited similar patterns in both the calibration and validation datasets (Figure 3). In the visible spectral region (400–710 nm), the *in situ* canopy reflectance was higher at low N supply, whereas in the near-infrared spectral region (70–950 nm), the canopy reflectance tended to decrease with decreasing N rates. In addition, reflectance in the near-infrared region led to greater variability compared with that in the visible region, the radiation of which chlorophyll absorbs. The results show that radiation in the near-infrared region was responsive to the different N supply during different growth stages but tended to saturate at high N supply.

### Correlation of the Leaf Nitrogen Concentration With *In situ* Canopy Spectra for the Calibration Datasets

**FIGURE 4:**
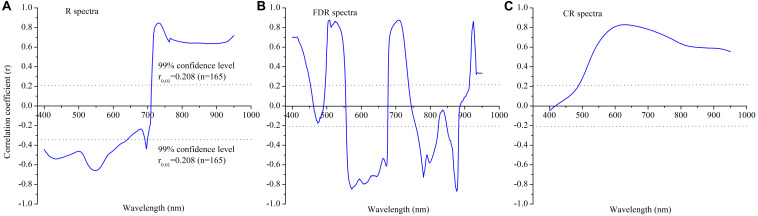
Correlation analysis between the leaf nitrogen concentration in winter wheat and canopy hyperspectral reflectance and its transformation in the calibration dataset under various N rates: **(A)** R spectra, **(B)** FDR spectra and **(C)** CR spectra.

To clarify the relationships of the canopy raw spectra and their subsequent transformation (*via* the FDR and CR techniques) with the winter wheat leaf N concentration, correlation analysis was applied to 551 spectral bands from 400 to 950 nm for the calibration datasets (Figure 4). First, a negative correlation was detected for the visible wavelengths, with the lowest r in the 525–570 nm range (*r* < 0.60, *n* = 165), whereas a positive correlation was found in the near-infrared region, with the greatest *r* value in the 720–745 nm region (*r* > 0.80, *n* = 165) for the raw spectra (Figure 4A). The FDR spectra showed a correlations throughout the full wavelength region (400–950 nm) that was stronger than the region of the raw spectra and exhibited more prominent valleys and peaks, for example, at 508, 525, 572, 709, 780, 876, and 925 nm, etc., (Figure 4B). Moreover, the leaf N concentrations exhibited a weak negative correlation with the CR spectra in the ultraviolet wavelength region (400–420 nm) and a strong positive relationship in the visible near-infrared wavelength region (420–950 nm). The average *r* values in the ultraviolet, visible, and near-infrared regions were −0.021, 0.551, and 0.636, respectively (Figure 4C).

### Accuracy of Leaf Nitrogen Concentration Estimation With the Multiple Linear Regression, Principal Component Regression, Partial Least Square, and Support Vector Machine

The robustness and accuracy of the four regression methods for leaf N concentration estimation using the statistical indicators *r*^2^_*cal/val*_, RMSE_*cal/val*_, and RPD_*cal/val*_ based on different spectral transformation techniques (raw, FDR and CR spectra) were evaluated and compared (Table 3). The FDR based on the PLS analysis generally yielded a measurement accuracy (*r*^2^_*cal*_ = 0.886, RPD_*cal*_ = 2.973; *r*^2^_val_ = 0.857, RPD_val_ = 2.535) that was greater than that of the other three methods and models. Moreover, the second most important technique for leaf N concentration estimation after the FDR-PLS was FDR-SVM, which yielded relatively low *r*^2^_*cal/val*_ (1.96 and 1.78%, respectively) and RPD_*cal/val*_ (3.69 and 6.38%, respectively) values in the calibration and validation datasets, respectively. The poorest performance was acquired using the FDR-MLR model (Table 3), with an *r*^2^_*cal*_ of 0.784, RPD_*cal*_ of 2.086, *r*^2^_val_ of 0.746, and RPD_val_ of 1.584, respectively. The aforementioned results indicate that the FDR-PLS model estimated the leaf N concentration the best and was identified as the preferred model for use in subsequent analyses.

**TABLE 3:** MLR, PCR, PLS, and SVM for predicting leaf nitrogen concentration in winter wheat based on different spectral transformation techniques.

Methods	Spectral techniques	Calibration datasets			Validation datasets		
		*r*^2^_cal_	RMSE_cal_	RPD_cal_	*r*^2^_val_	RMSE_val_	RPD_val_
MLR	Raw Spectra	0.706	0.699	1.731	0.681	0.824	1.542
	**FDR Spectra**	**0.784**	**0.580**	**2.086**	**0.746**	**0.802**	**1.584**
	CR Spectra	0.753	0.631	1.918	0.731	0.792	1.604
PCR	Raw Spectra	0.764	0.585	2.068	0.755	0.631	2.013
	**FDR Spectra**	**0.837**	**0.501**	**2.415**	**0.811**	**0.575**	**2.209**
	CR Spectra	0.814	0.522	2.318	0.793	0.603	2.106
PLS	Raw Spectra	0.816	0.518	2.336	0.806	0.601	2.113
	**FDR Spectra**	**0.886**	**0.407**	**2.973**	**0.857**	**0.501**	**2.535**
	CR Spectra	0.854	0.463	2.613	0.824	0.576	2.205
SVM	R Spectra	0.804	0.573	2.112	0.789	0.624	2.035
	**FDR Spectra**	**0.869**	**0.422**	**2.867**	**0.842**	**0.533**	**2.383**
	CR Spectra	0.831	0.501	2.415	0.807	0.604	2.103

*The FDR is highlighted to emphasize the best predictive performance of the four models for predicting leaf nitrogen concentration. MLR, multiple linear regression; PCR, principal component regression; PLS, partial least square; SVM, support vector machine.*

### Effective Wavelength Identification

#### Number of Latent Variables and Cross-Validation

**FIGURE 5:**
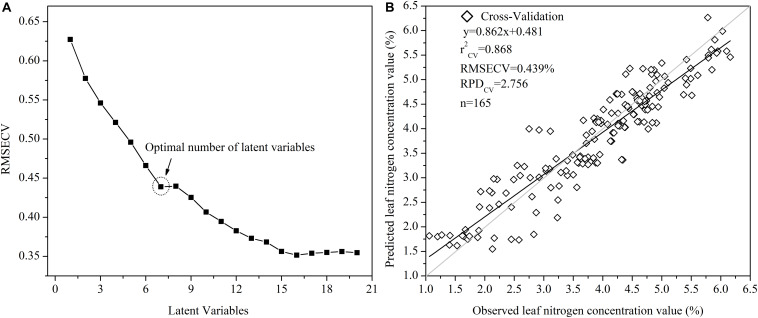
Plots with the root mean square error of cross-validation (RMSECV) with the number of latent variables **(A)** and cross-validation between the observed and predicted leaf nitrogen concentration values **(B)**.

In this study, the ONLVs was first determined according to the lowest value of the RMSECV (Figure 5A) *via* the leave-one-out method based on the FDR-PLS model (Figure 5B). To identify the ONLVs, the calibration datasets were applied to investigate how well the model with a different number of latent variables fit the data. As shown in Figure 5A, when the number of latent variables increased, the RMSECV value of the FDR-PLS model tended to decrease. However, the presence of inadequate latent variables led to underfitting, showing the requirement of a balance between the RMSECV value and the number of latent variables. With this criterion, the ONLVs for the FDR-PLS was 7. Figure 5B shows the accuracy of the cross-validation based on the FDR-PLS for the leaf N concentration estimation. Compared with those from the best fit technique (FDR) for the PLS method, both the *r*^2^ (*r*^2^_CV_ = 0.868) and RPD (RPD_CV_ = 2.756) values in the cross-validation were relatively high, indicating that the model was acceptable and that the ONLVs was suitable.

### Loading Weights of the First-Derivative Reflectance–Partial Least Square Regression Model

**FIGURE 6:**
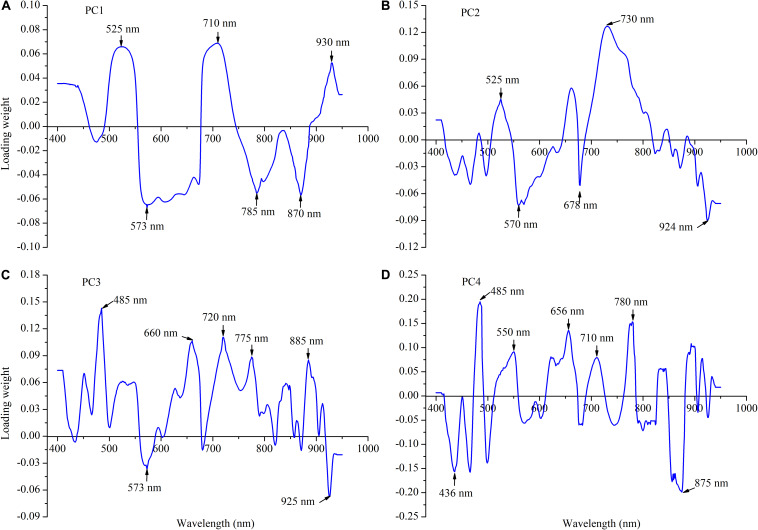
Loading weight over the full wavelength regions for the first four latent variables (PC1–PC4) of the first-derivative reflectance (FDR)-partial least square (PLS) for leaf nitrogen concentration estimation: **(A)** PC1, **(B)** PC2, **(C)** PC3, and **(D)** PC4.

To first clarify the relative impact of each wavelength, the loading weight value was computed and analyzed on the basis of the FDR-PLS model output (Figure 6). The loading weights showed how the latent variables resulting from the FDR-PLS model were developed from scaled estimators of the *in situ* reflectance spectra (Figures 5, 6). A relatively high absolute loading weight value indicates that the specific wavelength is crucial for the estimation of the leaf N concentration of winter wheat. In this study, the first four latent variables elucidated more than 81% of the canopy spectral reflectance variances and 82% of the leaf N concentration variances. The highest absolute loading weight values of each wavelength for rapidly estimating the leaf N concentration were in the visible (525 and 573 nm), red-edge (710 nm), and near-infrared wavelengths region (785, 870, and 930 nm) in the first latent variables of the FDR-PLS model (Figure 6). The selected wavelengths of the second loading weight were also in the visible (525 and 570 nm), red-edge (678, and 730 nm), and near-infrared wavelengths region (930 nm), while those of the third and fourth loading weights were nearly the same as those of the first and second loading weights.

### Effective Wavelength Identification by Variable Importance in Projection Values Based on the First-Derivative Reflectance–Partial Least Square Model

**FIGURE 7:**
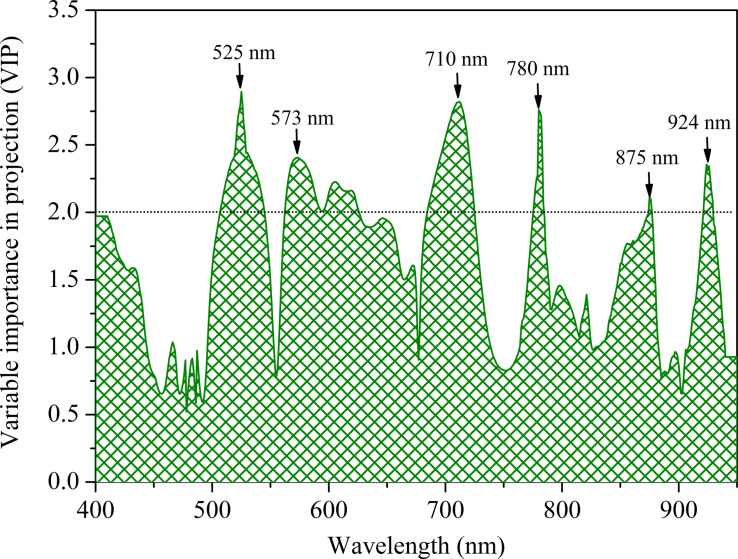
The variable importance in projection (VIP) values over the full wavelength regions for leaf N concentration estimation.

Owing to the high dimensionality of canopy spectral reflectance data with redundancy between the adjacent wavelengths, it was necessary to select several effective wavelengths that have the most representative information for rapidly estimating the leaf N concentration of winter wheat. Thus, the VIP scores were applied to select the effective wavelengths for predicting the leaf N concentration from the full spectral region on the basis of the FDR-PLS model (Figure 7). Generally, a high VIP score shows that the specific wavelength is of vital importance (the threshold value of VIP is 1.0). As shown in Figure 7, given that numerous wavelengths have relatively high VIP scores (>1.0), it was difficult to identify and distinguish the effective wavelengths for rapidly estimating the leaf N concentration. Therefore, the threshold value of the VIP was set at 2.0 in this study, and six wavelengths were selected as effective, two in the visible (525 and 573 nm), one in the red-edge (710 nm), and three in the near-infrared region (780, 875, and 924 nm) (Figure 7). Obviously, the identified effective wavelengths based on the VIP for estimating the leaf N concentration were shown in the same region of PC1 loading weight (Figure 6A).

### Multiple Linear Regression, Principal Component Regression, Partial Least Square, and Support Vector Machine Analysis With Effective Wavelengths

**FIGURE 8:**
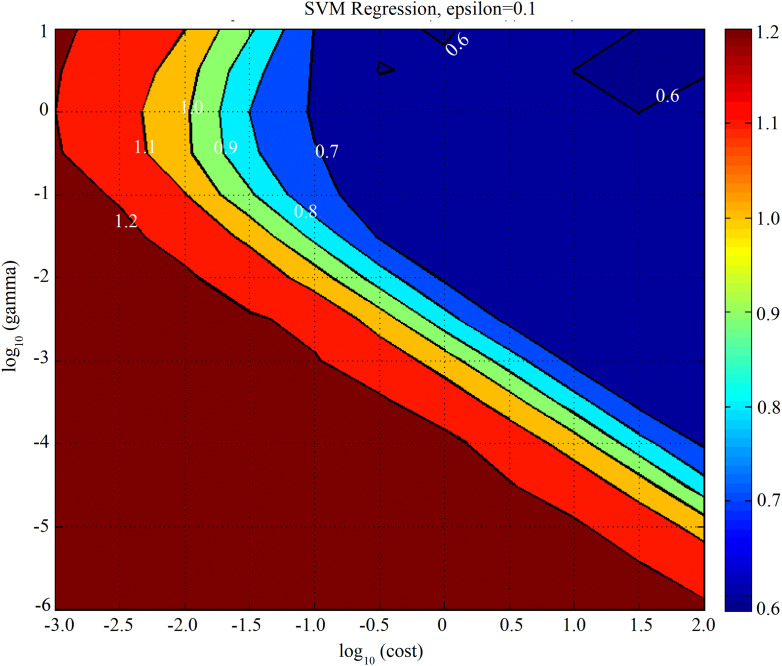
Contour map view of *g, c*, selected by the grid-search method with the first-derivative reflectance (FDR)–support vector machine (SVM) model for leaf nitrogen concentration prediction.

To further illuminate the potential and robustness of the identified effective wavelengths for rapidly estimating the leaf N concentration of winter wheat, MLR, PCR, PLS, and SVM analyses were developed again based on these effective wavelengths. Figure 8 shows the optimal setting of the meta-parameters with the FDR-SVM model. In this study, the optimal combination of epsilon (0.1), *g* (3.16), and *c* (100) were calculated based on the RMSECV. Moreover, the results shown in Table 4 indicated that the FDR-PLS model not only exhibited better performance on the calibration datasets, but also offered higher prediction accuracy on the validation datasets. Although 98.91% (551 vs. 6) of the canopy spectral reflectance variable information was eliminated for the leaf N concentration estimation, the *r*^2^_val_ (4.6% for FDR-PLS and 2.3% for FDR-SVM) and RPD (13.5% for FDR-PLS and 4.5% for FDR-SVM) values only showed a slight reduction. The results showed that the identified effective wavelengths and selected revalidated models were promising for rapidly estimating the leaf N concentration of winter wheat with less computational effort.

**TABLE 4:** FDR-MLR, FDR-PCR, FDR-PLS, and FDR-SVM analysis for leaf nitrogen concentration prediction of winter wheat using effective wavelengths.

Models	Calibration datasets			Validation datasets		
	*r*^2^_cal_	RMSE _cal_	RPD _cal_	*r*^2^_val_	RMSE _val_	RPD _val_
FDR-MLR	0.753	0.642	1.885	0.713	0.895	1.419
FDR-PCR	0.808	0.581	2.083	0.784	0.615	2.065
FDR-PLS	0.854	0.452	2.677	0.819	0.569	2.232
FDR-SVM	0.857	0.458	2.642	0.823	0.557	2.280

## Discussion

Our study demonstrates that CSRS can successfully estimate winter wheat N status. Canopy reflectance decreased from visible region and increased from near-infrared region as leaf N concentration increased (Figure 3). Increased N rates could increase greenness, reflecting a combination of chlorophyll concentration, structural agronomic parameters, and leaf anatomical structure characteristics (Colwell, 1974; Yao et al., 2013; Barankova et al., 2016). The interpretation of the effect of canopy spectral reflectance followed the general assumption that a decrease in greenness or chlorophyll creates higher reflectance in the visible region due to decreased light absorption from a lower chlorophyll concentration (Hinzman et al., 1986; Li et al., 2018a, b). The opposite effect was found in the near-infrared region; the relative high reflectance spectra observed were due to the canopy and leaf internal scattering and the apparent structure parameters, such as leaf area index and biomass (Colwell, 1974; Hansen and Schjoerring, 2003).

In general, two major approaches have been developed for remote estimation of crop N status: (i) empirical statistical methods and (ii) physically based retrieval methods such as canopy radiative transfer models (RTMs) (Darvishzadeh et al., 2011; Li et al., 2016). These two methods are mutually complementary (Viña et al., 2011), however, the main disadvantages in using RTM is the ill-posed nature of model inversion (Combal et al., 2002; Houborg et al., 2009), meaning that the inverse solution is not always unique as various combinations of canopy parameters may yield almost similar spectra (Fang et al., 2003). Consequently, the empirical approaches are used more extensively than RTM due to their straightforward mechanisms and efficient computations and have been proposed as better predictors of crop N status (Viña et al., 2011; Delegido et al., 2013). Our study also demonstrates that CSRS can successfully estimate winter wheat N status using the multivariate statistical approaches. Results indicated that the FDR-PLS was superior to the other models in estimation accuracy (Table 3). These results are consistent with those of Wang et al. (2017) and Nguyen and Lee (2006), showing that the PLS method could reduce the high dimensionality and multicollinearity problem of spectral data. The PCR method accounts for only the variance of the explanatory independent variables without considering the internal relationships between the wavelengths and response dependent variables, whereas the PLS accounts for both (Atzberger et al., 2010). Although MLR is the most widely used method for crop biophysical and biochemical estimation using spectral method (Curran et al., 2001; Wang et al., 2011), it might have a collinearity problem (Grossman et al., 1996).

To further rank the estimation models in their predictive power, we calculated several other statistical parameters like the slope (b), intercept (a), and coefficient of deviation (CD) of the linear regressions (*y* = *a* + *b*x) (Supplementary Table S2). The CD values are all higher than 1.0, indicating that the four models overestimated the predicted values compared to the measured data (Müller et al., 2008). The reason for this is presumably the higher range of the calibration datasets than the validation datasets (Table 2), which can be subject to the field conditions uncertainty and as a result lead to considerably overestimation error in the modeling process.

In this study, we mainly discussed the effect of the visible near-infrared range of canopy reflectance on winter wheat N estimation. For a typical crop canopy, reflectance in the visible spectrum and near-infrared region is often used to estimate leaf N concentration indirectly due to the strong positive correlation with leaf chlorophyll content and pronounced sensitivity to canopy structures (Kokaly, 2001; Gitelson et al., 2005; Li et al., 2019). However, the sensitive absorption wavelength of N lies in short-wave-infrared, which is easily obscured by water-vapor absorption characteristics (Curran, 1989; Fourty et al., 1996; Feng et al., 2008). Moreover, the spectral sensing information obtained in this study was from field-based spectral radiometers; research on aerial-based hyperspectral imagery for crop N status estimation is an important tendency in precision agriculture (Nigon et al., 2015; Bruning et al., 2019). Unlike conventional field-based spectrometers that only collect spectral information for a single point, a hyperspectral imaging system can obtain images of the whole target for each wavelength recorded (Wu and Sun, 2013). Future work should evaluate these results for diverse species, under different environmental conditions, and using not only *in situ* measurements but also airborne and satellite data.

## Conclusion

The overall results from this research showed that winter wheat leaf N concentration could be assessed with reasonable accuracy from field *in situ* canopy spectral data. We conclude that: (i) the FDR-PLS regression model was performed better than other model techniques evaluated for leaf N concentration of winter wheat; (ii) Six bands centered at 525, 573, 710, 780, 875, and 924 nm were identified as effective wavelengths for leaf N concentration estimation using the *in situ* canopy spectra; (iii) An acceptable accuracy with r^2^ (*r*^2^_*cal*_ = 0.857; *r*^2^_val_ = 0.823) and RPD (RPD_*cal*_ = 2.642; RPD_val_ = 2.280) was acquired using the FDR-SVM based on the effective wavelengths.

## Data Availability Statement

All datasets presented in this study are included in the article/Supplementary Material.

## Author Contributions

LL designed the study and wrote the manuscript. YW conceptualized the manuscript. DL and LY conducted the field experiments. JW contributed the tables and figures. All authors contributed to the article and approved the submitted version.

## Conflict of Interest

The authors declare that the research was conducted in the absence of any commercial or financial relationships that could be construed as a potential conflict of interest.
