# Supplementary material for: Multivariate Analysis Models Based on Full Spectra Range and Effective Wavelengths Using Different Transformation Techniques for Rapid Estimation of Leaf Nitrogen Concentration in Winter Wheat
Source: Front Plant Sci. 2020 Jun 26;11:755. doi: 10.3389/fpls.2020.00755 (PMC7333249; doi:10.3389/fpls.2020.00755)
Supplement: Supplementary file 1 [file Table_1.DOCX]

**Supplementary Material for**

**Sensitivity of Spectral Effective Wavelengths for Rapid Estimation of Leaf Nitrogen Concentration in Winter Wheat (*Triticum aestivum* L.) Using *in situ* Canopy Spectroscopy**

**Supplementary TABLE| S1** Seasons, cultivars, soil status, treatments, and sampling dates for the four field experiments.

| **Exp. No.** | **Seasons**  **and sites** | **Cultivars** | **Soil status** | **Treatments** | **Sampling dates** | **No. of samples** | **Data function** |
| --- | --- | --- | --- | --- | --- | --- | --- |
| Exp. 1 | 2017-2018  Yuanyang county | Pingan 11 | Type: fluvo-aquic soil, Organic-M: 17.9 g kg^-1^, Soil pH (CaCl_2_): 7.3, Total N: 1.02 g kg^-1^, Available P: 0.029 g kg^-1^, Available K: 0.145 g kg^-1^ | N rates (kg ha^-1^):  N0 (0), N90 (90), N180 (180), N270 (270), N360 (360) | Tillering/  Jointing/  Heading/  Mid-filling | 60 | Calibration |
| Exp. 2 | 2017-2018  Hebi city | Zhengmai 369 | Type: fluvo-aquic soil, Organic-M: 19.7 g kg^-1^, Soil pH (CaCl_2_): 7.4, Total N: 1.14 g kg^-1^, Available P: 0.029 g kg^-1^, Available K: 0.152 g kg^-1^ | N rates (kg ha^-1^):  N0 (0), N75 (75), N150 (150), N225 (225), N300 (300) | Tillering/  Jointing/  Heading/  Mid-filling | 60 | Validation |
| Exp. 3 | 2018-2019  Wenxian county | Pingan 11 | Type: fluvo-aquic soil, Organic-M: 18.4 g kg^-1^, Soil pH (CaCl_2_): 7.1, Total N: 1.06 g kg^-1^, Available P: 0.033 g kg^-1^, Available K: 0.159 g kg^-1^ | N rates (kg ha^-1^):  N0 (0), N60 (60), N120 (120), N180 (180), N240 (240), N300 (300) | Tillering/  Jointing/  Heading/  Initial-filling/  Late-filling | 90 | Validation |
| Exp. 4 | 2018-2019  Hebi city | Zhengmai 369 | Type: fluvo-aquic soil, Organic-M: 21.9 g kg^-1^, Soil pH (CaCl_2_): 7.4, Total N: 1.28 g kg^-1^, Available P: 0.035 g kg^-1^, Available K: 0.168 g kg^-1^ | N rates (kg ha^-1^):  N0 (0), N60 (60), N120 (120), N180 (180), N240 (240), N300 (300), N360 (360) | Tillering/  Jointing/  Heading/  Initial-filling/  Late-filling | 105 | Calibration |

**Supplementary TABLE| S2** Slopes (b), intercepts (a) of linear regressions (y=a+bx), CD of estimated values of leaf nitrogen concentration by FDR-MLR, FDR-PCR and FDR-PLS model to measured values derived from the validation datasets.

| Models | Calibration datasets | | |  | Validation datasets | | |
| --- | --- | --- | --- | --- | --- | --- | --- |
|  | b | a | CD |  | b | a | CD |
| FDR-MLR | 0.851 | 0.452 | 1.069 |  | 0.838 | 0.133 | 1.029 |
| FDR-PCR | 0.867 | 0.627 | 1.102 |  | 0.814 | 0.848 | 1.197 |
| FDR-PLS | 0.897 | 0.423 | 1.099 |  | 0.865 | 0.641 | 1.127 |

*Slope (b) and intercept (a) were tested for being significantly different from one and zero, respectively. CD is the coefficient of deviation, the values served as a measure of how much the derived values lie under or over the measured ones. It is scaled from zero with no upper limit, whereas one stands for no differences from measured to estimated values. The CD formula is as follows:*

$$CD=\frac{\sum_{j=1}^{n} {(x_{j}-\bar{x)}}^{2}}{\sum_{j=1}^{n} {(y_{j}-\bar{x)}}^{2}}$$

*where x_j_ are the measured values; y_j_ are the estimated values; n is the number of samples; and* $\bar{x}$ *is the mean of the measured data.*
